# The geometry of the vergence-accommodation conflict in mixed reality systems

**DOI:** 10.1007/s10055-024-00991-4

**Published:** 2024-04-11

**Authors:** Xiaoye Michael Wang, Daniel Southwick, Ian Robinson, Michael Nitsche, Gabby Resch, Ali Mazalek, Timothy N. Welsh

**Affiliations:** 1 Faculty of Kinesiology & Physical Education, University of Toronto, Toronto, ON Canada; 2 Synaesthetic Media Lab, Toronto Metropolitan University, Toronto, ON Canada; 3 Ivan Allen College of Liberal Arts, Georgia Institute of Technology, Atlanta, GA USA; 4 Faculty of Business and Information Technology, Ontario Tech University, Oshawa, ON Canada

**Keywords:** Virtual reality, Augmented reality, Vergence-accommodation conflict, Depth perception, Manual pointing

## Abstract

Mixed reality technologies, such as virtual (VR) and augmented (AR) reality, present promising opportunities to advance education and professional training due to their adaptability to diverse contexts. Distortions in the perceived distance in such mediated conditions, however, are well documented and have imposed nontrivial challenges that complicate and limit transferring task performance in a virtual setting to the unmediated reality (UR). One potential source of the distance distortion is the vergence-accommodation conflict—the discrepancy between the depth specified by the eyes’ accommodative state and the angle at which the eyes converge to fixate on a target. The present study involved the use of a manual pointing task in UR, VR, and AR to quantify the magnitude of the potential depth distortion in each modality. Conceptualizing the effect of vergence-accommodation offset as a constant offset to the vergence angle, a model was developed based on the stereoscopic viewing geometry. Different versions of the model were used to fit and predict the behavioral data for all modalities. Results confirmed the validity of the conceptualization of vergence-accommodation as a device-specific vergence offset, which predicted up to 66% of the variance in the data. The fitted parameters indicate that, due to the vergence-accommodation conflict, participants’ vergence angle was driven outwards by approximately 0.2°, which disrupted the stereoscopic viewing geometry and produced distance distortion in VR and AR. The implications of this finding are discussed in the context of developing virtual environments that minimize the effect of depth distortion.

## Introduction

In recent years, the advances in different digital technologies have fueled the development of various mixed reality (MR)^1^ technologies, especially virtual (VR) and augmented (AR) reality. Combined with its adaptability and flexibility for creating simulated environments, MR has been shown promise to be effective in training both basic cognitive skills (Chang et al. 2017, 2018; Makransky et al. 2019) and occupational skills (Barkokebas et al. 2019; Jeelani et al. 2017). Yet, despite recent developments and advancements, visual perception in virtual environments still presents issues that are not matched in the physical, unmediated reality (UR). These discrepancies are most noticeable with respect to the compression of perceived depth, a technical challenge that has not been solved since the 1990s (Kline and Witmer 1996; Witmer and Kline 1998; Witmer and Sadowski, 1998; see Renner et al. 2013 for a review). To gain a deeper insight into the challenges of depth perception in AR and VR, the present study used a manual pointing task to evaluate the effect of depth compression on visually guided actions in VR, AR, and UR. Using the behavioral results, a geometrical model was developed to account for characteristics of constant errors of pointing movements across all three modalities. The model showed a good fit and suggested that depth compression in VR/AR can be partially attributed to the vergence-accommodation conflict.

Depth perception, the ability to perceive distance to objects in the world, is an integral part of visual perception. Following the ecological framework formulated by Gibson (1986), the discourse on depth perception can be built on an evaluation of the physical properties of the environment-observer system (Koenderink and van Doorn 2008). In a physical environment, light reverberates between various surfaces and is structured by the unique properties of these surfaces. Observers pick up the structured light at a specific time and place, which specifies the lawful spatiotemporal relationship between observers and their surroundings. Because of the lawfulness that governs the interaction, every piece of visual information in the physical environment specifies the same spatial relationship. Although visual information can be delineated in various forms, the comparison of visual perception between UR and VR/AR commonly focuses on stereopsis and motion parallax. The current study examines the role of stereopsis and its related oculomotor mechanisms in depth perception across modalities through the investigation of manual interactions in peri-personal space. On the other hand, the effect of motion parallax, or the constant update of the patterns of light that observers receive as they navigate through the environment, is beyond the scope of the present study. A detailed discussion of motion parallax on 3D distance and direction perception beyond the peri-personal space can be found in Wang and Troje (2023).

Stereopsis originates from the binocular disparity, or the separation of the images, between the retinas as a result of the lateral separation between the two eyes (Cumming and DeAngelis 2001; Howard et al. 1995; Julesz 1964; Poggio and Poggio 1984). To digitally recreate stereopsis in MR systems, stereoscopic displays are used to present two slightly different images to each eye, mimicking the effect of binocular disparity. On a physiological level, the interpupillary distance (the distance between the pupils in each eye; IPD) mediates the distance specified by stereopsis due to the intrinsic perceptual geometry of the system. Using a telestereoscope, which leverages light refraction to manipulate the observer’s effective IPD, studies have shown changes in IPD affect the scaling of binocular disparity, where a larger IPD would result in shorter perceived distance and vice versa (Coats et al. 2014; Pan et al. 2014). In the case of MR systems, IPD corresponds to the distance between the two stereoscopic displays (or stereo base). Normally, the users would need to manually adjust the headset’s IPD to match their own IPD. The level of adjustment, however, can be limited (e.g., Quest 2 only offers three fixed IPD settings) and the IPD measurement could also be inaccurate (Hibbard et al. 2020). The consequence of mismatching IPDs in MR devices is akin to that of a telestereoscope, where the intended distance (Renner et al. 2015), object size (Kim and Interrante 2017), and even the size of one’s own virtual body (Mine et al. 2020) is inaccurately perceived. In other words, the inaccurate IPD of the stereo base could contribute to distortions of perceived depth in MR.

On a retinal level, the rendered images of stereoscopic MR systems faithfully reflect the projective geometry that underlies the lawful relationship between observers and their surroundings. However, when visual perception is mediated by a digital device, other aspects of the perceptual and non-perceptual processes are perturbed. For instance, studies have demonstrated the impact of the headset’s field of view (Buck et al. 2018; Kline and Witmer 1996; Masnadi et al. 2021) and weight (Buck et al. 2018; Willemsen et al. 2009), as well as the screens resolution (Jää-Aro and Kjelldahl 1997; Ryu et al. 2005) on distance perception in VR (see Kelly 2022 for a review). In addition to these hardware-specific challenges, the idea of using screens to approximate the perceptual geometry of the physical environment imposes a fundamental challenge to the human visual system, resulting in inconsistencies in what different types of visual information specify. Two relevant visual processes addressed in the present study are accommodation and vergence.

When fixating on an object in the physical environment, the curvature of the lens of each eye is changed so that the perceived object can be in focus on the retinal surface. The process of changing the curvature of the eye is termed *accommodation*. Simultaneously, the observer rotates the two eyes, either inwardly or outwardly, so that the projections of the object’s image fall in the center of both retinas. This process is termed *vergence*. Vergence works to facilitate the fusion of both images into a single image during perceptual processing that occurs in higher-order centers of the cerebral cortex. Due to the oculomotor nature of these processes (i.e., these changes result from motor commands to muscles of the eye), accommodation and vergence are categorized as *extra*-retinal information for visual perception, which contrasts with retinal information such as binocular disparity (Wexler and van Boxtel 2005). Despite their difference in nature, retinal and extra-retinal information are tightly intertwined, where the extra-retinal processes control how the information falls on the eye and vice versa (Fry 1983; Judge and Cumming 1986; Mays and Gamlin 1995; Morgan 1968; Schor and Ciuffreda 1983). The “blurring” of the images on the retina can drive the accommodative state of the lens, while the binocular disparity can drive the vergence angle between the two eyes. Additionally, there is also a crosslink between vergence and accommodation (Eadie et al. 2000; Hung 1992; Hung et al. 1996; Hung and Semmlow 1980), where vergence drives the accommodative response of the lens and vice versa.

While the retinal and extra-retinal information is typically consistent in a natural viewing condition, approximating the patterns of light through screens would inadvertently disrupt such consistency, perturbing the interconnections between the perceptual mechanisms that underlie different sources of visual information. In a virtual environment conveyed through systems such as a head-mounted display (HMD), the user will attempt to constantly focus their eyes on the screen, at a fixed distance away from the eyes. Because the screen is at a fixed distance, the eye’s lens does not need to adjust its shape to maintain the focus of virtual objects at different distances (i.e., accommodation is fixed). In contrast, vergence and binocular disparity tend to follow the expected patterns of observers’ gaze direction, specifying the constantly changing distance between the eyes and the fixation point. This discrepancy between the distance specified via accommodation and vergence is called the vergence-accommodation conflict (VAC) (Batmaz et al. 2022; Bingham et al. 2001; Hoffman et al. 2008; Mon-Williams and Tresilian 1999; Wann et al. 1995). Because VAC disrupts the coupling among different sources of visual information and their interactions, VAC not only generates viewer discomfort and fatigue (Hoffman et al. 2008; Lambooij et al. 2009) but, more importantly, also affects the efficiency of motor planning and control (Batmaz et al. 2022; Batmaz et al. 2023b), as well as distance perception in VR/AR (Bingham et al. 2001; Kramida 2015; Singh et al. 2018; Swan et al. 2015). As well, the fully screen-based MR technologies (VR and video passthrough AR) also form a stark contrast with the optical passthrough AR, such as Microsoft’s HoloLens. Using translucent materials with holographic displays, optical passthrough AR provides the users with unmediated access to the physical environment and the body of the user while interacting with digital assets. In this context, while the hologram is still rendered at a fixed distance to the eyes that is subject to the effect of VAC, optical passthrough AR could eliminate the challenges with VAC and input latency in VR and video passthrough AR because unmediated access to real objects and limbs of the user does not require additional processing to render these critical features. Therefore, the similarities and differences between UR, VR, and optical passthrough AR could further shed light on the effect of VAC on distance perception and targeted movements.

Numerous methods have been proposed to address VAC and most of them focus on developing hardware that allows the adjustment of the display’s focal distance (Kramida 2015). To the best of the authors’ knowledge, the repercussions of VAC on the *perceptual geometry* associated with HMDs have rarely been examined (Barrera Machuca and Stuerzlinger 2019; Singh et al. 2018; Swan et al. 2015). The current study was designed to understand the stereoscopic viewing geometry of MR, including the effect of VAC and inaccurate IPD of the stereo base, by comparing depth perception in UR, VR, and AR using a manual pointing task. According to Bingham and Pagano (1998), adopting a perception–action approach is necessary to study definitive distance perception because distance perception intrinsically requires spatial calibration, which can be achieved through action-based visual feedback. Hence, participants in the present study were asked to point to several target locations as quickly and accurately as possible in UR, VR, and AR. Using the endpoints of the movements as an index of perceived distance, systematic trends were identified between the perceived distance and target distance. Subsequently, a stereopsis-based geometrical model was developed that used an angular offset in the vergence angle to account for VAC. Finally, model fitting and comparison were performed to evaluate the effectiveness of the proposed model.

## Behavioral experiment

### Methods

#### Participants

Sixty adults completed the experimental protocols, with 20 in each modality (UR: 10 males and 10 females, aged between 19 and 31; VR: 8 males and 12 females, aged between 18 and 32; AR: 8 males and 12 females, aged between 18 and 30). Participants provided their full and informed consent prior to participation and received monetary compensation for their time. All participants were right-handed and had normal or corrected-to-normal vision. All procedures were approved and were consistent with the standards of the University of Toronto Research Ethics Board.

#### Stimuli and apparatus

Experimental sessions across all modalities were executed on a desktop computer with an Intel Core i7 CPU, 32 GB RAM, and an NVIDIA GTX 3080 GPU. For UR, the stimuli were presented on an Acer GD235HZ 24-inch monitor with a 1920 × 1080 resolution and 60 Hz refresh rate. For VR, the experiment was implemented and executed in Unity3D, a game engine software application for developing immersive environments. A custom virtual environment was constructed to replicate the physical environment of UR. The environment was presented via an HTC VIVE Pro Eye head-mounted display (HMD) with a resolution of 1440 × 1600 pixels per eye, a combined 110° field of view, and a refresh rate of 90 Hz. For AR, the stimuli were presented via a Microsoft HoloLens (2nd gen) holographic mixed reality glasses that have a 43° × 29° field of view with a holographic density of 2500 radiants (light points per radian) and a refresh rate of 60 Hz. HoloLens is an optical passthrough device with transparent lenses, allowing the user to see their physical surroundings in a natural viewing condition with holographic objects overlaid on top. Therefore, the effective field of view of the AR headset should equate to the participant’s natural field of view, which is slightly over 210° in a binocular viewing condition.

The participants’ pointing movements were recorded using an optoelectric motion tracking system (Optotrak, Northern Digital Inc., Waterloo, Ontario, Canada). This system recorded the three-dimensional (3D) coordinates of an infrared-emitting diode (IRED) at a 250 Hz sampling frequency. Before the beginning of each session, the experimenter would attach the IRED to the nail of the participant's right index finger using medical tape. Data collection was controlled using a custom Python-driven interface that communicates with the motion tracking system via the Optotrak Application Programmer’s Interface (OAPI).

**Fig. 1:**
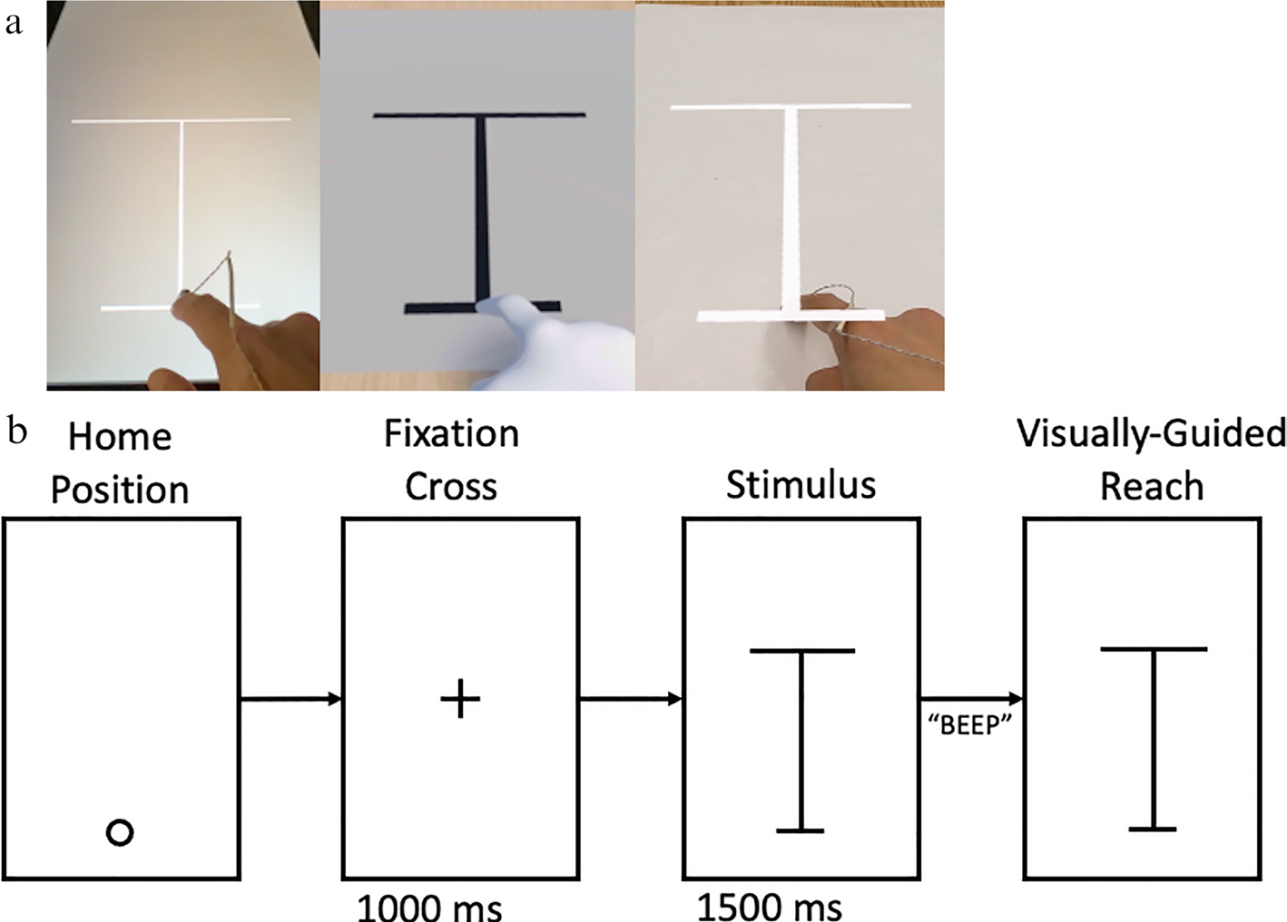
**a** Images of the pointing tasks performed in UR (left), VR (center), and AR (right) from the participant’s perspective. **b** A schematic illustration of the experimental setup and timeline for a single trial

Because different modalities rely on different hardware and each comes with its unique development environment and challenges, efforts were made to ensure a comparable experimental setup across modalities. For UR, the experiment was implemented and controlled via PsychoPy (Peirce 2007; Peirce et al. 2019). During the experiment, participants were seated in front of a table with a 24-inch monitor. The surface of the monitor was parallel to the tabletop. Participants sat at the short end of the monitor and reached the opposite side (away from the body along the mid-sagittal plane) during the experiment (Fig. 1a, left). To maintain consistency of visual information across conditions, participants were told to execute the aiming movements with a clenched fist except for the extension of the index finger. Participants had a full view of their own (real) hand. Movement trajectories of the tip of the right index finger were recorded using Optotrak and the single iRED on the tip of the index finger. Recordings were controlled directly through PsychoPy using the custom Optotrak Python interface.

For VR, participants sat in front of the same physical table as in the UR. Inside the virtual environment, a virtual table was rendered in front of the participants at the same height as the physical table to ensure haptic feedback was consistent with visual feedback. A flat, virtual surface was placed on the virtual table with the same dimensions as the monitor in UR. This virtual surface was used to render the experimental stimuli and instructions. The experiment was controlled via bmlTUX in Unity3D (Bebko and Troje 2020). One key challenge of VR is to accurately render the user’s effectors (e.g., hands) in the virtual environment in real time. Pilot testing showed that the HMD’s image-based hand-tracking capability with the two front-facing cameras was neither accurate nor reliable. Therefore, the more reliable and precise motion capture data from Optotrak was used to render and control a static, non-articulating virtual hand. Although there were slight differences in the orientation of the virtual to real hand, this virtual hand maintained the same posture as the participant’s real hand (i.e., clenched fist except for the extension of the index finger). The motion capture data collected via Python were streamed to Unity using a user datagram protocol (UDP) socket in real time. The temporal delay between Optotrak’s data acquisition and Unity3D’s rendering was small and below the refresh rate of the screens, with an average 2.74 ms lag (SD = 2.03). Because Optotrak and Unity3D use different coordinate systems, spatial calibration was performed. During the calibration, participants were instructed to turn on the HMD’s pass-through view to overlay the physical environment onto the virtual environment via the HMD’s two front-facing cameras. With the view of their physical hand, participants placed their right index finger (with an Optotrak IRED attached to the tip) sequentially at three virtual targets, forming a right triangle, on the surface of the virtual table. The positions of the virtual targets in the virtual space and the 3D locations of the participant’s index finger in the physical space were recorded and used to compute the transformation matrix (translation, rotation, and scale) that maps between the two spaces. Subsequently, this transformation was applied to the real-time positions from Optotrak to animate a virtual hand formed in a pointing gesture (Fig. 1a, center). Because a singular marker was used to track the 3D positions of the fingertip of the participant’s right index finger, the rendered virtual hand remained in a stationary pose. Despite only presenting a static hand in VR, participants all reported that they “felt like the hand was a part of them” and that they actually “own the hand”.

The AR experiment was also executed in Unity3D and used the same 3D assets as the VR experiment. Being an optical passthrough headset, HoloLens allowed the participants to simultaneously see the physical and virtual environments. In other words, the virtual environment and the virtual hand necessary for the VR condition were not required for AR. Instead, participants reached with and were able to see their own, physical hand (Fig. 1a, right). As noted in the figure, one of the drawbacks of overlaying virtual objects on top of the physical environment is the occlusion of the environment, including portions of the hand of the participant. Even though the stimulus was rendered to be on the tabletop, its image was holographically presented immediately in front of the participant’s eyes, which created the occlusion seen in the figure. Previous studies have shown that such occlusion only affects manual reaching performance using monocular vision, not binocular vision (Bingham et al. 2001). The communication between the AR glasses and Unity3D was achieved through Microsoft’s Mixed Reality Toolkit (MRTK) and Holographic Remoting. To minimize transmission lag, the headset was tethered to the desktop using a Thunderbolt 4 cable, purchased separately. At the beginning of each session, a QR code was used to anchor the virtual display at the center of the physical table, presented against a sheet of white paper.

#### Procedures and design

The experiment discussed in the current study is part of a larger research project (see Wang et al. (under review) for additional information). Figure 1b shows the experimental setup and procedures. For all modalities, participants sat in front of a table, 25 cm from the edge of the display, and rested their index finger at the home position 5 cm from the edge of the display before the start of each trial. Then, a fixation cross, 21 cm from the home position, appeared on the display for 1000 ms. The fixation cross was replaced by an I-like object (the stimulus) that appeared for 1500 ms. The initial home position coincided with the narrower end of the “I”. The participants were asked to immediately point to the farther, wider end of the “I” where the vertical line met the horizontal line. Participants were instructed to execute these movements as accurately and as quickly as possible after an acoustic beep signal. Participants’ movements were collected for 2500 ms, after which a visual prompt would appear asking the participants to return to the home position. There were three movement amplitudes (stimulus lengths of 20, 25, and 30 cm) presented in a random order. Each presentation of the 3 movement amplitudes was considered a block. Each block was repeated 16 times, and trials within each block were presented in a random order. The first block was used for familiarization and was not included in the subsequent data analysis, resulting in 45 (3 × 15) trials per participant. The original experiment also manipulated the perceptual availability of the target during the pointing movement and the shape of the wider end of the target, but the present report is restricted to the conditions in which the target was present throughout the movement and the target stimulus was presented as an “I”. Finally, as described earlier, the VR condition required an additional spatial calibration to allow participants to interact with the virtual environment, which was performed before each session.

#### Data reduction

To evaluate distance perception using movement data, the Trajectory Analysis Toolkit for Human Movement (TAT-HUM; an open-sourced Python toolkit focusing on human kinematic analysis; Wang and Welsh, 2024) was used to derive the pointing distance from the movement trajectories. The motion capture system was intentionally calibrated so that the primary movement direction (the direction in which the participants pointed) coincided with the z-axis of the system. Therefore, to derive the pointing distance, only the trajectory along the *z*-axis needs to be considered. Because pointing distance equates to the perceived length of the target, only using the distance along the primary axis, instead of the Euclidean distance on the horizontal plane, also helped to minimize the effect of extraneous lateral movement on the derived distance.

**Fig. 2:**
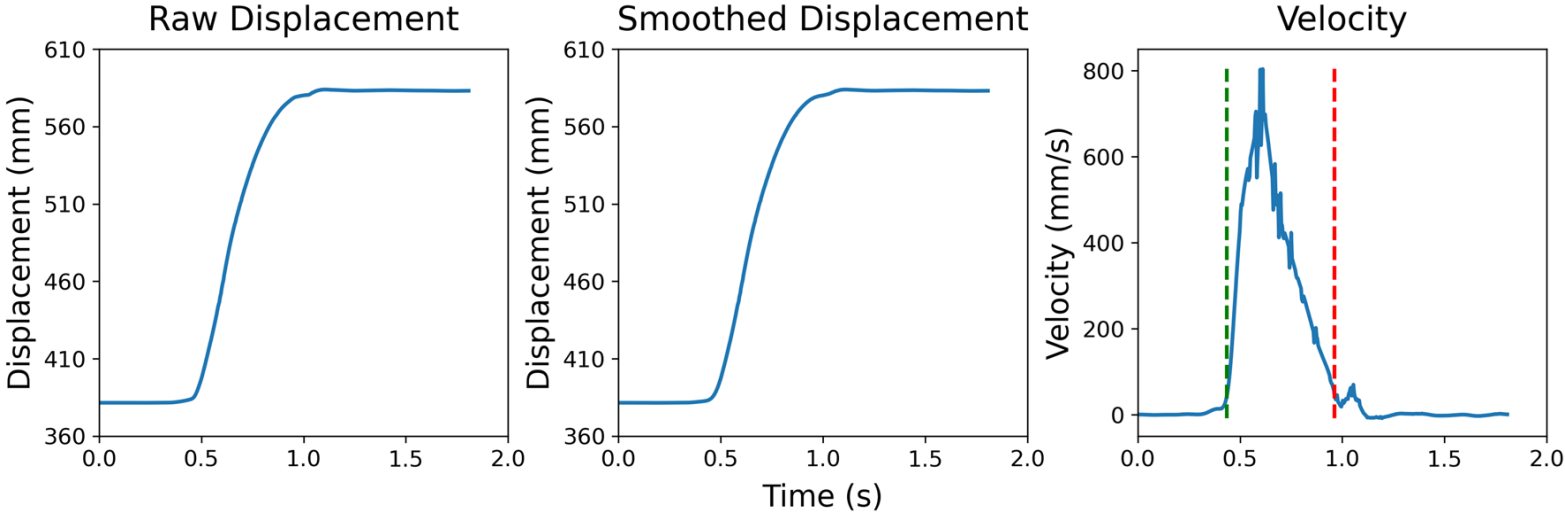
Illustration of the data reduction process. The raw displacement data were smoothed using a low-pass Butterworth filter. Then, a central difference method was used to derive the velocity. Movement initiation (green dashed line) and termination (red dashed line) were determined to be when the velocity exceeds or drops below a threshold of 50 mm/s (Color figure online)

Figure 2 illustrates the data reduction process. For each movement trajectory, displacement data was smoothed using a second-order low-pass Butterworth filter with a 250 Hz sampling frequency and a 10 Hz cutoff frequency. Subsequently, movement velocity was derived from the displacement data using a central difference method:

$$\frac{{dz_{{t_{n} }} }}{{dt_{n} }} = \left\{ {\begin{array}{*{20}l} {\frac{{z_{{t_{n + 1} }} - z_{{t_{n} }} }}{{t_{n + 1} - t_{n} }},} \hfill & {if\,\, n = 1} \hfill \\ {\frac{{z_{{t_{n + 1} }} - z_{{t_{n - 1} }} }}{{t_{n + 1} - t_{n - 1} }},} \hfill & {if\,\, 2 < n < N - 1} \hfill \\ {\frac{{z_{{t_{N} }} - z_{{t_{N - 1} }} }}{{t_{N} - t_{N - 1} }},} \hfill & {if\,\, n = N} \hfill \\ \end{array} } \right.$$

Movement onset and termination were determined with a velocity cutoff of 50 mm/s, where movement onset is when the velocity exceeds the threshold while movement termination is when the velocity drops below the threshold. Visually guided manual pointing movements commonly entail an initial ballistic phase that transports the hand to the vicinity of the target based on the perceived target distance and motor planning, and a second correction phase in which the observers use online proprioceptive and visual feedback, such as disparity matching (Bingham et al. 2023), to accurately arrive at the target (Khan et al. 2006; Woodworth 1899; see Elliott et al. (2010) for a review). While the ballistic phase tends to be a single, smooth movement with a parabolic velocity profile, the correction phase may even involve small and large velocity fluctuations that reveal the use of feedback to correct the ongoing movement. In some instances, the participant may overshoot the target and have to execute a reversal to land on the target. In other instances, the participant might initially undershoot the target and then execute an additional secondary sub-movement to achieve the target – such additional sub-movements are revealed when the hand’s velocity could even be reduced to near 0 and then increase again (e.g., see Fig. 2 Velocity subplot, where the velocity dropped below the threshold during the initial approach, but increased slightly afterward). Because the current study focuses on the effect of perturbed distance perception on manual pointing, emphasis was placed on the primary sub-movement movement which was identified when the velocity dropped below a hard velocity cutoff. Although the primary sub-movements are subject to correction (see Elliott et al. 2010; including those in the present study; see Wang et al. in preparation), the present analysis did not incorporate the secondary, discrete adjustments in the determination of the endpoint.

The reach distance is the difference in *z-*coordinates between the positions of the iRED (fingertip) at movement onset and termination, whereas the movement time (MT) is the time between movement onset and termination. To examine deviations of reach distance from target lengths, constant errors were computed and used for subsequent analysis. Constant error was defined as the difference between the real reach distance of the limb and the stimulus length (20, 25, and 30 cm). Note that any resulting differences between visual conditions in constant error could be attributed to the speed-accuracy tradeoff, where, for instance, faster and more variable movements could lead to an increase in constant error (Fitts 1954). Thus, differences in constant error across the UR, VR, and AR conditions might arise from a speed-accuracy trade-off (in which participants who emphasized accuracy might have a lower constant error and a lower variable error, but at the expense of longer MTs, while other participants who emphasized speed might have a higher constant error, higher variable error, and have shorter MTs) instead of due to differences that are inherent to the systems. To assess this potential, variable error and MT were also analyzed. Variable errors were calculated as the standard deviation of constant errors for each participant in each unique combination of conditions (i.e., mediation and target length).

#### Statistical analysis

A series of mixed-design analysis of variances (ANOVAs) was used to analyze the effect of mediation conditions (between-subject; UR, VR, AR) and stimulus lengths (within-subject; 20, 25, 30 cm) on constant error, variable error, and MT. Greenhouse–Geisser corrections, as indicated by the decimal values in the reported degrees of freedom, were applied to effects that violate the sphericity assumption. Significant main and interaction effects were further evaluated using post-hoc Tukey’s pairwise comparisons.

### Results and discussion

**Fig. 3:**
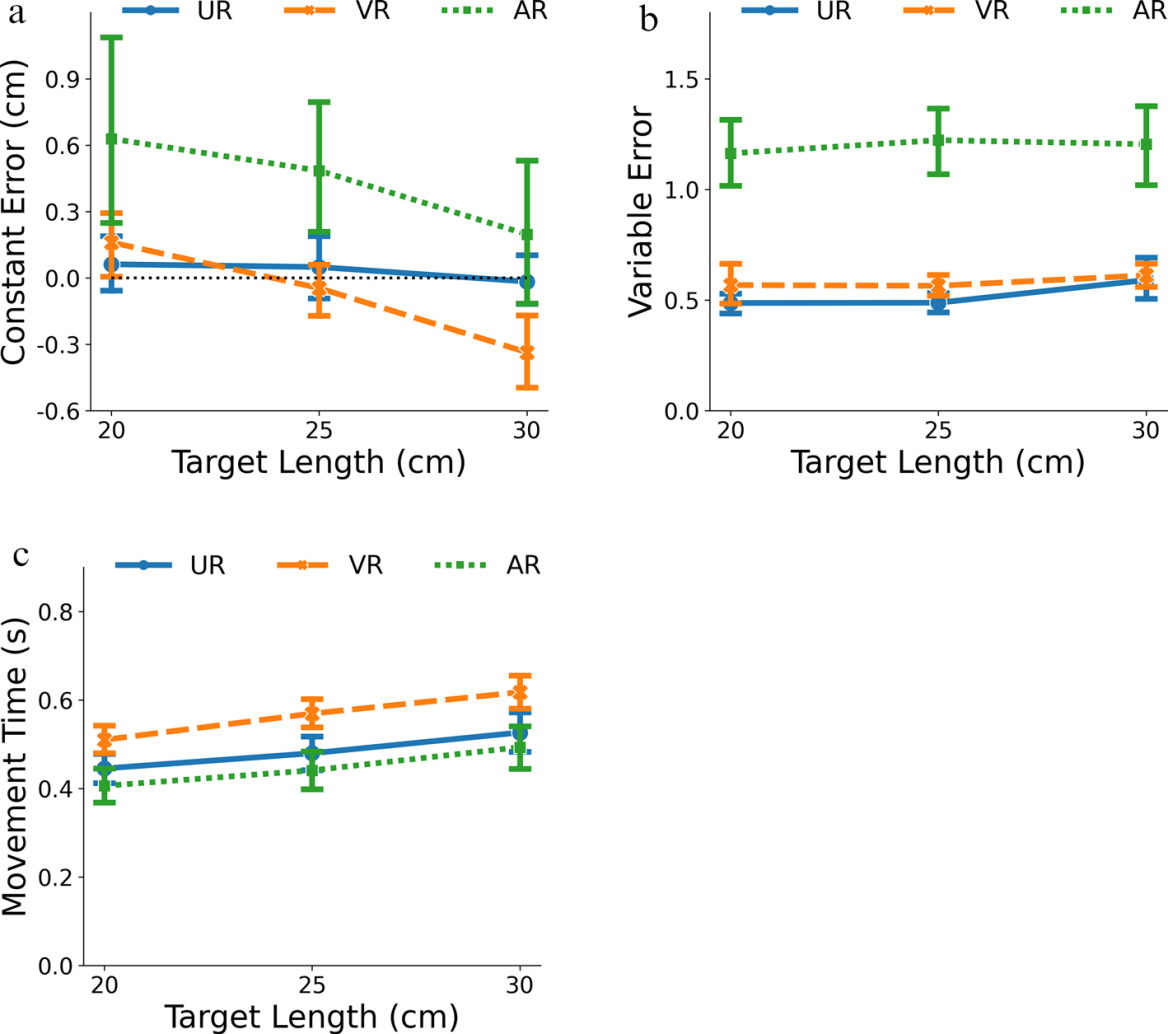
Mean **a** constant errors, **b** variable errors, and **c** movement time (MT) for each modality as a function of target length. Lines represent constant errors for different modalities whereas error bars represent the 95% confidence intervals

The ANOVA on constant error showed a significant main effect of mediation, $F\left(2, 57\right)=5.72, p<0.01, {\eta }_{p}^{2}=0.17$, and of target length, $F\left(2, 114\right)=29.43, p<0.001, {\eta }_{p}^{2}=0.34$. There was also a significant interaction between mediation and target length, $F\left(4, 114\right)=4.37, p<0.01, {\eta }_{p}^{2}=0.13$. For the main effect of mediation, post-hoc pairwise comparison showed that constant error in AR was significantly greater than that in UR (mean difference = 0.41, SE = 0.16, *p* < 0.05) and VR (mean difference = 0.51, SE = 0.16, *p* < 0.01) and there was no difference between UR and VR (mean difference = 0.11, SE = 0.16, *p* = 0.79). As Fig. 3a shows, there was a noticeable overestimation of distance in AR (95% CI = [0.16, 0.72]), whereas the mean errors for UR and VR were not different from 0 (UR: 95% CI = [− 0.25, 0.31]; VR: 95% CI = [-0.35, 0.20]).

Figure 3a also reveals that the effect of target length could be largely attributed to the variations of constant error in the VR and AR conditions, but not the UR condition. This observation is consistent with the significant interaction between mediation and target length. Therefore, instead of performing separate post-hoc analyses for the main effect of target length, simple contrasts for each modality with sequential comparisons for different target lengths (i.e., 25–20 cm, and 30–25 cm) were performed to minimize the number of planned comparisons. For UR, neither target length pair yielded a significant difference (25–20 cm: mean difference =  − 0.01, SE = 0.075, *p* = 0.98; 30–25 cm: mean difference =  − 0.07, SE = 0.068, *p* = 0.56). For VR, both comparisons were significant (25–20 cm: mean difference =  − 0.21, SE = 0.075, *p* < 0.05; 30–25 cm: mean difference =  − 0.29, SE = 0.068, *p* < 0.001), suggesting a negative relationship between the constant errors and target length. Finally, for AR, the comparison between 25 and 20 cm was not significant (mean difference =  − 0.14, SE = 0.075, *p* = 0.11), but the comparison between 30 and 25 cm was (mean difference =  − 0.29, SE = 0.068, *p* < 0.001), suggesting a similar negative trend as in VR.

Analysis of variable error only showed a significant main effect of mediation, ${\text{F}}\left(2, 57\right)=72.98, p<0.001, {\eta }_{p}^{2}=0.72$. Neither target length (${\text{F}}\left(2, 114\right)=1.56, p=0.21, {\eta }_{p}^{2}=0.027$) nor the interaction between target length and mediation (${\text{F}}\left(4, 114\right)=0.51, p=0.73, {\eta }_{p}^{2}=0.018$) were significant. As Fig. 3b shows, the AR condition (mean = 1.20, 95% CI = [1.09, 1.31]) had larger variable error than the UR (mean = 0.52, 95% CI = [0.41, 0.63], *p* < 0.001) and VR (mean = 0.58, 95% CI = [0.47, 0.69], *p* < 0.001) conditions, while there was no difference between UR and VR (*p* = 0.60). The noticeable difference between AR and the other mediation conditions could be attributed to the intermixing of virtual assets with the physical environment in this AR condition. First, the virtual object was projected over the hand with the impact that the vision of the hand was disrupted during movement execution. Second, although a QR code was used to anchor the virtual assets in the physical environment, drifting in the HoloLens’s signal may still occur (Guinet et al. 2019; Johnson et al. 2022), resulting in an inconsistent relationship between the physical (i.e., the participant’s hand) and the virtual (i.e., the target), while the recording of the location of the tip of the participant’s finger (via iRED) was maintained.

For MT, the effects of mediation, ${\text{F}}\left(2, 57\right)=8.75, p<0.001, {\eta }_{p}^{2}=0.24$, target length, $F\left( {1.63, 92.98} \right) = 308.97, p < 0.001, \eta_{p}^{2} = 0.84$, and their interaction, ${\text{F}}\left( {3.26, 92.98} \right) =$ $3.25, p<0.05, {\eta }_{p}^{2}=0.10$, were all significant. As the target length increases, MTs were longer to move to the target location, which accounts for the main effect of length (Fitts 1954). More interestingly, the main effect of mediation revealed that MT in the VR condition (mean = 0.57 s, 95% CI = [0.52, 0.62]) was significantly greater than MT in UR (mean = 0.48 s, 95% CI = [0.43, 0.54], *p* < 0.05) and AR (mean = 0.45, 95% CI = [0.40, 0.50], *p* < 0.001) conditions. The difference between UR and AR was not significant (*p* = 0.42). Because HoloLens 2 in the AR condition is an optical passthrough device, the participants perceived the movement of their physical hand in the physical environment, just like in the UR condition. This consistency in the visual information of the hand explains the consistency in MTs in the UR and AR conditions. In contrast, the VR condition completely removed the participants from the physical environment on a perceptual level, and the novelty of 3D interactions in a virtual environment (Liu et al. 2009), as well as the input latency (Teather et al. 2009), could have led to an increased MT. That is, although the real and virtual hands were in the same orientation and same (general) space and delays in visual feedback about the locations of the real and virtual hand were negligible, participants were not observing their “real” hand in these conditions. Finally, for the significant interaction, post-hoc pairwise comparison showed significant differences between consecutive target lengths (i.e., 25–20 cm and 30–25 cm) for all three mediation conditions. As Fig. 3c shows, there was no discernable difference across mediation conditions for different target lengths.

Overall, the analysis of variable error and MT showed differences between different mediation conditions, but the results are not what would be predicted by a speed-accuracy trade-off. Participants in VR had variable errors that were not different from their peers in UR but had longer MTs than those in UR, whereas participants in the AR condition had MTs that were not different from their peers in UR but had higher variable errors than those in UR. Given that speed-accuracy trade-offs do not seem to capture differences between UR and VR/AR, the discussion will now focus on the constant error analysis which is the main measure that assesses the potential differences in perception.

The comparison among the three modalities revealed one noticeable pattern: While UR yielded very accurate movements with consistent constant error across different target lengths close to 0, constant errors in the mediated conditions (i.e., VR and AR) were shorter as target length increased. Because target length is proportional to the viewing distance and constant error is a measure of distance perception, this negative trend suggests that the perceived depth becomes more compressed as the distance between the target and the observer becomes larger. Importantly, because there was a lack of consistent difference in MT and variable error across conditions in each target distance, this negative relationship between constant error and target distance observed in VR and AR (Fig. 3a) could potentially reflect a common underlying mechanism that affects distance perception.

To identify this mechanism, it is crucial to evaluate the common elements between VR and AR. Using a closed-loop manual pointing task in which participants had a constant vision of their hand and the target, the participants could use real-time online visual feedback to correct their movements. Because participants could control the end of their movement and use visual feedback to complete the movement and to ensure (their perceived) accuracy, any inaccuracy in performance could be attributed to factors that could be device/modality-specific. For instance, the VR condition completely removed the participants from their physical environment, requiring them to control a virtual, disembodied though “connected” hand using their actual hand to point at the target. Evaluated alone, this setup contains numerous features that may potentially affect performance accuracy, such as disembodiment (Gonzalez-Franco et al. 2019; Mohler et al. 2010), the discrepancy between participants’ virtual and actual hands (Linkenauger et al. 2013), and, specific to online motor control, motion-to-photon latency (Warburton et al. 2022). In contrast, the AR condition with optical pass-through allowed participants to interact with virtual targets with their real hands while still having unperturbed visual access to the entire physical environment. In this case, none of the aforementioned issues related to controlling the virtual hand in VR would apply to AR. Therefore, if similar negative trends in VR and AR share a common mechanism, such a mechanism could originate from a common *perceptual* feature.

In the context of visually-guided targeted action, binocular disparity provides crucial visual information for successful targeted actions (Bingham et al. 2001, 2023). As discussed in the Introduction, one common hardware feature shared across stereoscopic displays – as in the HMDs for VR and AR – is the vergence-accommodation conflict (VAC), which could affect depth perception with stereopsis (Batmaz et al. 2022; Hoffman et al. 2008; Mon-Williams and Tresilian 1999; Wann et al. 1995). The question, then, is how exactly does VAC affect distance perception? Or, more pertinently, how could VAC be used to predict the behavioral outcome depicted in Fig. 3a. To examine this issue, a geometrical model was developed to explore the perceptual geometry in this process.

## A geometrical model of the vergence-accommodation conflict

### Model description^2^

#### Model environment

**Fig. 4:**
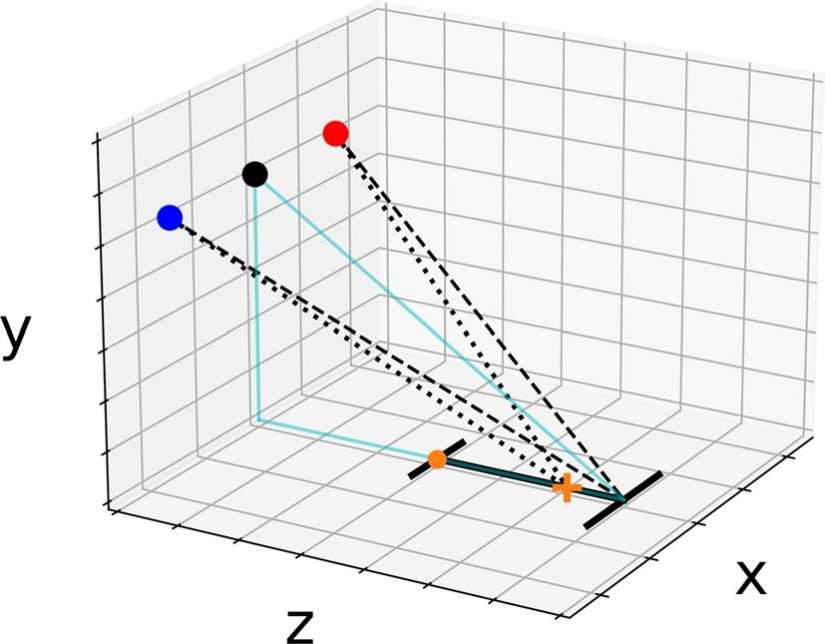
An illustration of the model 3D environment. The target was plotted on the *xz*-plane, where the orange dot on the left indicates the home position and the target’s starting point. The red and blue dots indicate the left and right eyes, respectively, whereas the black dot represents the cyclopean eye. The dotted lines start at each eye and end at the fixation point (orange cross), indicating the fixation angle. Similarly, the dashed lines converge at the target endpoint. The cyan right triangle was used to derive the perceived distance, where its hypotenuse was derived based on Eq. (3) (Color figure online)

Figure 4 shows the setup of the model environment where a 3D Cartesian coordinate system was established. Similar to the motion capture system’s frame of reference, the *xz*-plane represents the horizontal plane with the *z*-axis corresponding to the direction of egocentric depth. The *y*-axis denotes the vertical direction. The home position (orange dot) was at a distance ${{\text{D}}}_{{\text{home}}}$ (= 30 cm) from the origin:

$${{\text{P}}}_{{\text{home}}}\left(0, 0, {{\text{D}}}_{{\text{home}}}\right)$$

The fixation cross (orange cross) was always ${{\text{D}}}_{{\text{fix}}}$ (= 21 cm) away from the home position:

$${{\text{P}}}_{{\text{fix}}}\left(0, 0, {{\text{D}}}_{{\text{home}}}+{{\text{D}}}_{{\text{fix}}}\right)$$

Given that the starting point of the movement coincides with the home position, for a target of length ${\text{L}}$, its end point’s position is:

$${{\text{P}}}_{{\text{end}}}\left(0, 0,\mathrm{ D}_{{\text{home}}}+{\text{L}}\right)$$

To delineate the perceptual process, it is important to distinguish between the geometry based on which the images are rendered (rendering side) and that which the observers use to recover the location of the target’s endpoint (perceptual side), which will be discussed separately.

#### Rendering side

On the rendering side, the center of projection of the HMD needs to be specified. Assuming the eyes are H above the origin and let the HMD’s IPD be ${{\text{IPD}}}_{{\text{HMD}}}$, the locations of the two virtual cameras based on which the HMD’s images are rendered can be specified as:

$${{\text{HMD}}}_{{\text{left}}}\left(-\frac{{{\text{IPD}}}_{{\text{HMD}}}}{2},H, 0\right)$$

$${{\text{HMD}}}_{{\text{right}}}\left(\frac{{{\text{IPD}}}_{{\text{HMD}}}}{2},H, 0\right)$$

${{\text{IPD}}}_{{\text{HMD}}}$ was set at a constant 62.5 mm to reflect the headset’s IPD during the experiment. Using a fixed IPD was to mimic the inaccurate IPD adjustment that normal, everyday consumers would encounter, which would help to develop a more generalizable model. Then, the vergence angle, or the angle corresponding to the participants’ fixation point (between the two dotted lines in Fig. 4), can be derived as:

1 $$\phi = {\text{arccos}}\left( {\frac{{\left( {{\text{HMD}}_{{{\text{left}}}} - {\text{P}}_{{{\text{fix}}}} } \right) \cdot \left( {{\text{HMD}}_{{{\text{right}}}} - {\text{P}}_{{{\text{fix}}}} } \right)}}{{\left\| {{\text{HMD}}_{{{\text{left}}}} - {\text{P}}_{{{\text{fix}}}} } \right\|\left\| {{\text{HMD}}_{{{\text{left}}}} - {\text{P}}_{{{\text{fix}}}} } \right\|}}} \right)$$

Similarly, the angle corresponding to the target endpoint (between the two dashed lines in Fig. 4) is:

$$\tau_{{{\text{end}}}} = {\text{arccos}}\left( {\frac{{\left( {{\text{HMD}}_{{{\text{left}}}} - {\text{P}}_{{{\text{end}}}} } \right) \cdot \left( {{\text{HMD}}_{{{\text{right}}}} - {\text{P}}_{{{\text{end}}}} } \right)}}{{\left\| {{\text{HMD}}_{{{\text{left}}}} - {\text{P}}_{{{\text{end}}}} } \right\|\left\| {{\text{HMD}}_{{{\text{left}}}} - {\text{P}}_{{{\text{end}}}} } \right\|}}} \right)$$

Combining the two, the binocular disparity of the endpoint, as specified by the HMD, is:

2 $$\delta_{{{\text{end}}}} = \phi_{{{\text{HMD}}}} - \tau_{{{\text{end}}}}$$

Note that binocular disparity is calculated in line with the approach used by Bingham and colleagues (e.g., Anderson and Bingham 2010; Coats et al. 2014). An alternative approach, where the disparity is the difference between the angles formed between the fixation point, the left/right eye, and the target, would yield a disparity of 0° due to the symmetrical setup.

#### Perceptual side

On the perceptual side, the observers need to recover the spatial information using the rendered geometry from the HMD. If the perceptual geometry is identical to the rendering geometry, then the spatial information could be accurately and unequivocally recovered. Unfortunately, two key factors, VAC and IPD differences, may affect this assumption. Due to VAC, the effectiveness of the disparity information could be affected. Following the approach taken by Swan et al. (2015) and Singh et al. (2018), VAC could be modeled as a constant offset, ${\beta }_{{\text{offset}}}$, to the vergence angle, which yields the perceptual fixation angle $\widehat{\phi }$:

$$\hat{\phi } = \phi + \beta_{{{\text{offset}}}}$$

Then, to derive the perceptual target angle, ${\widehat{\tau }}_{{\text{end}}}$, which will be subsequently used to obtain the perceived endpoint location, the disparity provided by the HMD could be subtracted from the perceptual fixation angle following the same relationship in Eq. (2):

$${\widehat{\tau }}_{{\text{end}}}=\widehat{\phi }-{\delta }_{{\text{end}}}$$

To convert ${\widehat{\tau }}_{{\text{end}}}$ to distance, IPD is needed. On the perceptual side, the observers’ IPD, instead of the HMD’s IPD, should be used. Compared to the default ${{\text{IPD}}}_{{\text{HMD}}}$, observers’ IPDs may be different (due to anatomical differences), resulting in slightly different eye positions than those based on which the disparity in the display was produced. Although modern VR HMDs allow users to adjust the IPD, the adjustment method is somewhat crude (e.g., by turning a knob on the side of the headset to adjust the distance between the two lenses for HTC VIVE) and could be inaccurate (e.g., Oculus Quest 2 only has three IPD presets). Nevertheless, these adjustment methods all require independent measurement of the IPD and, due to not being a part of the experimental protocol, were not provided to participants. Therefore, to capture this effect, a different IPD, ${{\text{IPD}}}_{{\text{par}}}$, was used to describe the perceptual geometry. Given ${{\text{IPD}}}_{{\text{par}}}$, the distance between the observers’ cyclopean eye and the target endpoint, as specified in the perceived target endpoint angle, ${\widehat{\tau }}_{{\text{end}}}$, is:

3 $$\hat{d}_{{{\text{cyclo}}}} { = }\frac{{{\text{IPD}}_{{{\text{par}}}} {/2}}}{{{\text{tan}}\left( {\hat{\tau }_{{{\text{end}}}} {/2}} \right)}}$$

This distance represents the hypotenuse of the right triangle (cyan lines) formed between the cyclopean eye, the target endpoint, and the origin. Therefore, the Pythagorean theorem can be used to derive the modeled perceived target length, $\widehat{l}$:

$$\widehat{l}=\sqrt{{\widehat{d}}_{{\text{cyclo}}}^{2}-{H}^{2}}-{D}_{{\text{home}}}$$

In its present form, the model requires two unknowns, the constant vergence offset, ${\beta }_{{\text{offset}}}$, and the participant’s IPD, ${{\text{IPD}}}_{{\text{par}}}$, to derive the modeled perceived target length, $\widehat{l}$. To evaluate how well the model predicts the behavioral results, an optimization process was used to identify the optimal parameter values that minimize the difference between $\widehat{l}$ and the perceived target length $l$ from the experiment. To prevent target length, $L$, from taking too much variance in the model fitting process, constant errors were used in the objective function:

$$\epsilon =l-L\mathrm\,\,{ and }\,\,\widehat{\epsilon }=\widehat{l}-L$$

Thus, the model fitting process entails minimizing the difference between $\widehat{\epsilon }$ and $\epsilon$:

$$\mathop {\text{arg min}}\limits_{{\beta_{{{\text{offset}}}} ,{\text{ IPD}}_{{{\text{par}}}} }} \sum \left[ {\left( {\hat{\epsilon } - \epsilon } \right)^{2} } \right]$$

#### Model comparison

Given the model setup and the fitting strategy, it is important to determine the allocation of the unknown parameters for the data from each modality. Theoretically, the focal distances of the lenses in different devices (and, therefore, modalities) are different, meaning that different devices should have their unique vergence offsets, and the offsets should be the same across different participants within the modality. In contrast, ${{\text{IPD}}}_{{\text{par}}}$ should be unique to each participant given various anatomical differences. According to Dodgson (2004), the average IPD for adults is 63.36 mm (SD = 3.83) and can generally range from 52 to 78 mm. Therefore, there should be one fitted parameter for $\beta_{{{\text{offset}}}}$ and 20 fitted parameters for ${{\text{IPD}}}_{{\text{par}}}$, resulting in a total of 21 fitted parameters for each modality with 20 participants. This version of the model is theoretically driven and is considered as the baseline.

From a model-fitting standpoint, it is also important to devise alternative models to which the baseline model could be compared, which, in turn, could provide further theoretical insights into the model’s validity. To this end, alternative versions of the baseline model are introduced. For instance, since the baseline model only contains one ${\beta }_{{\text{offset}}}$ for each modality, it would be interesting to see how introducing a unique ${\beta }_{{\text{offset}}}$ for each participant would affect the model fit. In other words, there would be participant-specific ${{\text{IPD}}}_{{\text{par}}}$ and $\beta_{{{\text{offset}}}}$, resulting in a total of 40 fitted parameters for each modality, each with 20 participants. Obviously, including more parameters could increase the model’s goodness of fit as measured by ${r}^{2}$. However, the measure of the model’s quality, specifically, the Bayesian Information Criterion (BIC) could reveal whether the trade-off between a better fit and more parameters is worthwhile as BIC also takes into consideration the number of parameters. Theoretically, if this alternative version provides a better fit and a higher quality than the baseline model, the hypothetical vergence offset should be the result of the interaction between the headset’s unique optical properties and the individual observer’s unique perceptual system.

Another alternative is to not include $\beta_{{{\text{offset}}}}$ in the fitting process at all. This alternative is especially important for UR because the participants simply interacted with the physical environment that did not elicit VAC. In this case, there would only be 20 ${{\text{IPD}}}_{{\text{par}}}$ for each participant. Again, in the physical environment, the model’s hypothetical ${{\text{IPD}}}_{{\text{HMD}}}$ should be equal to the participants’ IPD as visual perception was not mediated. Because the current model setup requires an initial value for ${{\text{IPD}}}_{{\text{HMD}}}$, if the above conjecture is true, then the resulting ${{\text{IPD}}}_{{\text{par}}}$ from this version should be at around the default ${{\text{IPD}}}_{{\text{HMD}}}$, suggesting a lack of effect of the IPD discrepancy for UR (since there is none). Therefore, if the model in its general form is a suitable description for all modalities, the version without ${\beta }_{{\text{offset}}}$ should fit the UR better than the others.

Finally, in the last variation, each participant would have a unique vergence offset value but not a unique IPD. In other words, during model fitting, the ${{\text{IPD}}}_{{\text{par}}}$ in Eq. (3) is simply replaced with the default ${{\text{IPD}}}_{{\text{HMD}}}$. The inclusion of this variation is to examine the relative importance of ${\text{IPD}}_{{{\text{par}}}}$. Compared to the baseline version (1 $\beta_{{{\text{offset}}}}$ and 20 ${\text{IPD}}_{{{\text{par}}}}$), if this alternative provides a better or similar fit, then the $\beta_{{{\text{offset}}}}$ alone can pick up the variance in the data, suggesting overlapping effects of $\beta_{{{\text{offset}}}}$ and ${\text{IPD}}_{{{\text{par}}}}$, which may challenge the conceptual validity of the model.

Overall, four variations of the model are evaluated for each modality:

1. 1 $$\beta_{{{\text{offset}}}}$$ and 20 $${\text{IPD}}_{{{\text{par}}}}$$
2. 20 $$\beta_{{{\text{offset}}}}$$ and 20 $${\text{IPD}}_{{{\text{par}}}}$$
3. No $$\beta_{{{\text{offset}}}}$$ and 20 $${\text{IPD}}_{{{\text{par}}}}$$
4. 20 $$\beta_{{{\text{offset}}}}$$ and no $${\text{IPD}}_{{{\text{par}}}}$$ (s $${\text{IPD}}_{{{\text{par}}}} = {\text{IPD}}_{{{\text{HMD}}}}$$)

To examine and compare the effectiveness of different variations of this model in predicting the behavioral constant errors (Fig. 3), the data were split into a training and a testing set with a 7:3 ratio. Specifically, using a fixed random seed (for reproducibility), 70% of the trials for each unique combination of mediation and target length were randomly sampled for each participant (10 repetitions × 3 target length × 20 participants = 600 data points) and are used to fit the model. Then, the remaining 30% of the data (5 repetitions × 3 target length × 20 participants = 300 data points), in conjunction with the fitted parameters, was used to evaluate the model’s predictive ability. Finally, because of the relatively large between-trial variability, the behavioral data were averaged across repetitions for the test and train sets, respectively, to derive one mean measure for the analysis. Therefore, for each mediation condition, the training and testing set each contained 3 (target length) × 20 (participants) = 60 data points.

Model fitting was performed using SciPy’s (Virtanen et al. 2020) *curve_fit()* function, which uses non-linear least squares with the Levenberg–Marquardt algorithm (Moré, 1978) that optimizes parameter values to fit an arbitrary function to data. To evaluate the model’s goodness of fit, $r^{2}$ and BIC are calculated for both the training and testing sets. $r^{2}$ provides information on the proportion of variance that the fitted model could explain in the experimental data, while BIC captures whether the model is the *true* model for the data, where the model with a smaller BIC is preferred. To compute BIC for a least-square fit, a simplified formula was used:

$${\text{BIC}} = n\ln \left( {\frac{{{\text{SSE}}}}{n}} \right) + k\ln \left( n \right) + n\ln \left( {2\pi } \right) + n$$

where ${\text{SSE}}$ is the sum of squared errors between the model prediction and behavioral results, $n$ is the number of observations used for the fit, and $k$ is the number of estimated parameters in the model.

### Model fitting results

Table 1 shows the model goodness-of-fit measures for different modalities and different versions of the model. Overall, the model provides a good fit for the data. For UR, the $r^{2}$ values are generally comparable across different versions. Unsurprisingly, the version with the most parameters (20 $\beta_{{{\text{offset}}}}$, 20 ${\text{IPD}}_{{{\text{par}}}}$) yielded the highest $r^{2}$ for the training set. However, different models’ fit for the testing set was equivalent, between 0.41 and 0.44. More importantly, BIC showed that despite the better fit, the (20 $\beta_{{{\text{offset}}}}$, 20 ${\text{IPD}}_{{{\text{par}}}}$) model was penalized for having too many parameters, resulting in much higher BIC scores for both the training and testing sets. Among the remaining versions, the baseline (1 $\beta_{{{\text{offset}}}}$, 20 ${\text{IPD}}_{{{\text{par}}}}$) yielded the lowest BIC for the training data but the second highest for the test, suggesting that it may not be suitable to predict performance in UR. In comparison, the other two versions, (No $\beta_{{{\text{offset}}}}$, 20 ${\text{IPD}}_{{{\text{par}}}}$) and (20 $\beta_{{{\text{offset}}}}$, no ${\text{IPD}}_{{{\text{par}}}}$), generated similar BIC scores for the test set where that of the former was slightly lower.

**Table 1:** Goodness-of-fit comparison for the four variations of the model for each modality using the train and test data sets

	UR				VR				AR			
	$r^{2}$		BIC		$r^{2}$		BIC		$r^{2}$		BIC	
	Train	Test	Train	Test	Train	Test	Train	Test	Train	Test	Train	Test
1 $\beta_{{{\text{offset}}}}$, 20 s ${\text{IPD}}_{{{\text{par}}}}$	0.76	0.43	29.51	89.44	**0.74**	**0.66**	**60.33**	**101.79**	**0.87**	**0.44**	**120.25**	**205.65**
20 $\beta_{{{\text{offset}}}}$, 20 ${\text{IPD}}_{{{\text{par}}}}$	0.86	0.41	74.30	168.83	0.86	0.61	99.80	187.94	0.97	0.34	102.80	292.74
No $\beta_{{{\text{offset}}}}$, 20 ${\text{IPD}}_{{{\text{par}}}}$	**0.73**	**0.44**	**32.09**	**84.12**	0.53	0.42	93.04	129.69	0.84	0.34	129.98	211.12
20 $\beta_{{{\text{offset}}}}$, no ${\text{IPD}}_{{{\text{par}}}}$	0.74	0.43	29.76	84.83	0.52	0.40	93.89	130.96	0.85	0.36	124.25	209.51

$r^{2}$ represents the proportion of the variance in the behavioral data that is explained by the model. The higher the $r^{2}$, the better the fit. The Bayesian information criterion (BIC) evaluates the model’s goodness-of-fit but is different from $r^{2}$ because it also accounts for the number of parameters in the model. The lower the BIC, the more appropriate the model. Bolded rows indicate the version that provides the best fit for each modality

**Fig. 5:**
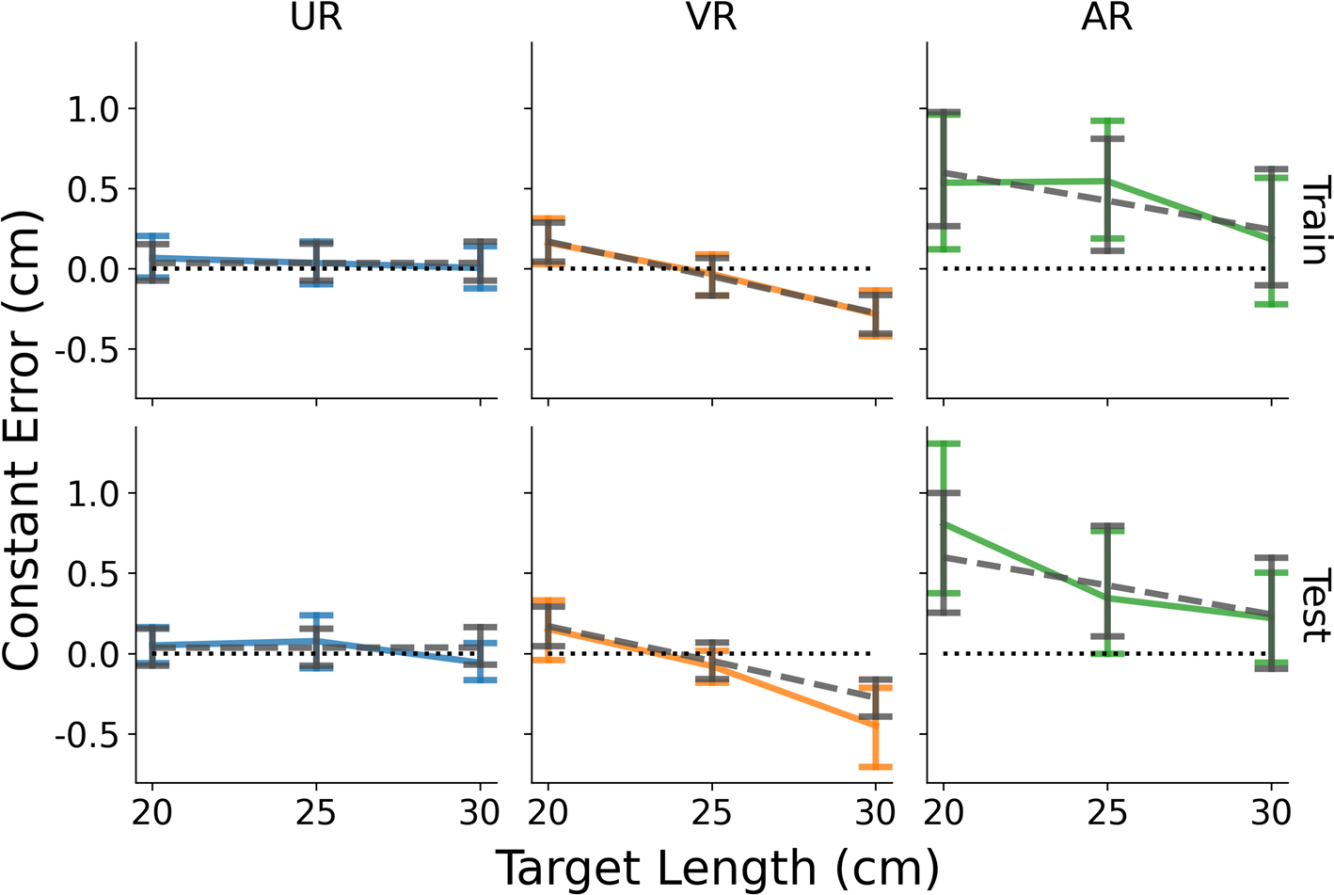
Behavioral (solid lines) and model (gray, dashed lines) results for constant errors (cm) as a function of stimulus length for different modalities (columns) and fitting types (top row: training set; bottom row: testing set). Only the optimal version of the model was used for each modality, as indicated in Table 1. Error bars represent 95% confidence intervals

As discussed earlier, because the perception in UR was not mediated by a screen or any virtual content, using $\beta_{{{\text{offset}}}}$ to describe performance in this condition is not exactly appropriate. Furthermore, although the (No $\beta_{{{\text{offset}}}}$, 20 ${\text{IPD}}_{{{\text{par}}}}$) version generated 20 unique IPDs for each participant, their values should equate to the hypothetical ${\text{IPD}}_{{{\text{HMD}}}}$ if the model’s conceptualization is correct. This is, in fact, the case: The mean fitted IPD value was 62.52 mm (SD = 0.17) and was not significantly different from the default ${\text{IPD}}_{{{\text{HMD}}}}$ of 62.50 mm (${\text{t}}\left( {19} \right) = 0.59, p = 0.56, 95\% {\text{CI}} = \left[ {62.44, 62.60} \right]$). Therefore, from the modeling, practical, and theoretical standpoints, the (No $\beta_{{{\text{offset}}}}$, 20 ${\text{IPD}}_{{{\text{par}}}}$) version of the model describes the data in UR the best. Figure 5 (left column) compares the behavioral results against the model predictions (based on the (No $\beta_{{{\text{offset}}}}$, 20 ${\text{IPD}}_{{{\text{par}}}}$) version) for the train and test sets, demonstrating a tight fit between the two. Overall, UR’s model fit results can be considered as a control. Although the model was originally designed to describe performance in the mediated conditions, its underlying perceptual geometry is correct and could be generalized to the unmediated condition and produce interpretable results.

More interesting findings reside in the mediated conditions. For VR, Table 1 reveals that the two versions that work well with UR, (No $\beta_{{{\text{offset}}}}$, 20 ${\text{IPD}}_{{{\text{par}}}}$) and (20 $\beta_{{{\text{offset}}}}$, no ${\text{IPD}}_{{{\text{par}}}}$), failed to fit the training and test data as well as the other alternatives in terms of $r^{2}$ and thus will not be considered. For the remaining two, the version with more parameters, (20 $\beta_{{{\text{offset}}}}$, 20 ${\text{IPD}}_{{{\text{par}}}}$), produced a better fit for the training data ($r^{2}$ = 0.86) than the baseline version (1 $\beta_{{{\text{offset}}}}$, 20 ${\text{IPD}}_{{{\text{par}}}}$; $r^{2}$ = 0.74), but a slightly lower $r^{2}$ with the testing data ($r^{2}$ = 0.61 vs. $r^{2}$ = 0.66). Furthermore, with fewer parameters, the baseline implementation also had much lower BIC values for both training (BIC = 60.33) and testing (BIC = 101.79) than the alternative version (BIC = 99.80 for training and BIC = 187.94 for testing). Overall, evidence suggests that the baseline version of the model, namely a device-specific vergence offset value across all participants and a unique IPD value for each participant, could adequately predict behavioral results, accounting for up to 66% of the variance in the constant errors of the testing data set.

Using the (1 $\beta_{{{\text{offset}}}}$, 20 ${\text{IPD}}_{{{\text{par}}}}$) version, Fig. 5 (middle column) compares the behavioral results with model predictions. Like the UR, the model could successfully predict the constant errors in the testing set. Regarding the fitted parameters, the mean IPD values were at 65.38 mm (SD = 0.18), which was significantly different from the default ${\text{IPD}}_{{{\text{HMD}}}}$, ${\text{t}}\left( {19} \right) = 71.02, p < 0.001, 95\% {\text{CI}} = \left[ {65.30, 65.47} \right]$. However, compared to the variability of the IPD on a population level (SD = 3.83; Dodgson 2004), that of the fitted IPDs was much lower. Because the present study did not explicitly measure participants’ IPD values, comparing the participants’ actual IPD with those from the model is not possible. However, it is likely that the lack of variability in the fitted IPD values could be attributed to the model setup, which will be discussed in detail in the General Discussion.

The more interesting finding, however, is the fitted value for the vergence offset, $\beta_{{{\text{offset}}}}$, which was 0.22°. One way to interpret the meaning behind this number is through the perceptual geometry depicted in Fig. 4. Given the fitted IPD values for each participant, their default fixation angle, $\phi$, could be easily determined based on Eq. (1), which is subsequently offset by a positive constant, $\beta_{{{\text{offset}}}}$, due to the VAC of the HMD. Then, using the same trigonometric relationship described in (3), the effective fixation distance because of VAC can be derived:

$$\hat{d}_{{{\text{fix}}}} = \frac{{{\text{IPD}}_{{{\text{par}}}} /2{ }}}{{\tan \left( {\left( {\phi + \beta_{{{\text{offset}}}} } \right)/2} \right)}}$$

Given the experimental setup, the unperturbed fixation distance on the rendering side was 71.41 cm, whereas the average fixation distance on the perceptual side was 68.49 cm (SD = 0.008). In other words, due to the VAC, the observers effectively fixated 2.93 cm *closer* than that specified by the perceptual geometry, which contributes to the depth compression observed in the present study.

Finally, for AR, model comparison revealed a similar pattern to VR. The two model versions with only a single parameter type, (No $\beta_{{{\text{offset}}}}$, 20 ${\text{IPD}}_{{{\text{par}}}}$) and (20 $\beta_{{{\text{offset}}}}$, no ${\text{IPD}}_{{{\text{par}}}}$), produced relatively higher $r^{2}$ for the training data, but fell short with the testing set. The version with too many parameters, (20 $\beta_{{{\text{offset}}}}$, 20 ${\text{IPD}}_{{{\text{par}}}}$), also suffered from a similar drawback: It could almost perfectly fit the training data ($r^{2}$ = 0.97) but had a mediocre performance with the testing set ($r^{2}$ = 0.34). In comparison, the model’s baseline implementation, (1 $\beta_{{{\text{offset}}}}$, 20 ${\text{IPD}}_{{{\text{par}}}}$), was able to fit both the training and testing data well ($r^{2}$ = 0.87 and $r^{2}$ = 0.44, respectively), and it also produced the smallest BIC score for the testing data. In sum, the model comparison suggests that, like VR, one could also describe the depth distortion in AR as a result of a device-specific vergence angle offset due to VAC. In terms of the fitted parameters, the derived IPD values were similar to those in VR (mean = 65.14 cm, SD = 0.53), and were significantly different from the default IPD used for model fitting, $t\left( {19} \right) = 21.71, p < 0.001, 95\% {\text{ CI}} = \left[ {64,89, 65.39} \right]$. Lastly, the vergence angle offset was found to be at 0.18°. With the same unperturbed fixation distance of 71.41, the average effective fixation distance was 69.01 (SD = 0.019), indicating an effective fixation distance of 2.41 cm closer than that specified by the rendering geometry.

## General discussion

The present study used the endpoints of movements from a manual pointing task to compare distance perception in UR, VR, and AR. Using a fixed velocity threshold, kinematic analysis extrapolated the endpoint to reflect the perceived distance of the target during motor planning to filter out any contribution from online correction. The difference between the target movement distances and the distances of the actual movements was leveraged as the index of the accuracy of perceived distance. Behavioral results showed a difference between VR and AR in terms of constant and variable errors as well as MT were observed. This finding is in contrast to an earlier study that did not report any difference between VR and AR in a similar pointing task (Batmaz et al. 2019). This difference between studies could be attributed to the different experimental setups. While the present study presented the targets on a tabletop against visible texture, Batmaz et al. (2019) presented the targets in the air. The presence of the tabletop provides not only additional distance information (i.e., texture gradient) for visually guided pointing movement (Bingham et al. 2022; Herth et al. 2021), but also somatosensory information about target and limb position via touch and proprioceptive senses. In VR, both the tabletop and the target were virtual whereas in AR, the virtual target was presented against a physical tabletop. This difference might have contributed to the difference between the two modalities that was not observed in Batmaz et al. (2019).

Importantly, behavioral results also indicated an increased compression in perceived depth as a function of distance for VR and AR, but not UR. A model based on the binocular viewing geometry was proposed to account for these results. Utilizing a constant vergence angle offset and participant-specific IPD values, the model could fit and predict the behavioral data well for all modalities. The fitted parameters suggest that, due to VAC in VR and AR, there was an approximately 0.2° constant inward offset in the fixation angle that effectively draws the fixation 2 to 3 cm closer than the distance specified by the rendering geometry. Because of this offset, targets were perceived to be increasingly closer to observers as the targets got farther away.

The primary inspiration for this model came from Swan et al. (2015) and Singh et al. (2018), who compared distance perception in AR and the physical environment using a depth matching task (see also Barrera Machuca and Stuerzlinger (2019) who modeled the effect of VAC in a Fitts’s Law task as the focal plane difference of targets at different depths). These studies used a collimated AR display that renders the virtual object at optical infinity (i.e., infinite focal depth). The authors found a systematic *overestimation* in the matched distance of 0.5 to 4 cm and suggested that this display characteristic forced the observers to rotate their eyes outwards by a constant amount, resulting in the observed depth expansion in their results. In the current study, the fixation distance was assumed to be 71.42 cm (see footnote 5 for derivation). For the VR headset (VIVE Pro Eye), although there was a lack of official documentation, studies, and other communications have indicated a focal distance of around 70 cm, including, 65 cm^3^ (Batmaz et al. 2023a), 70 cm (Krajancich et al. 2020), and 75 cm (Yeh et al. 2021). Although the numbers are inconsistent, it could be assumed that the headset’s focal distance was closer than the fixation distance, drawing the vergence angle inward and producing a positive vergence offset. Interestingly, Batmaz and colleagues (Batmaz et al. 2023a; Batmaz et al. 2023b) used the VIVE headset to render the target object at the headset’s focal distance (65 cm), which effectively mitigated the effect of VAC. Indeed, under this viewing condition, the participants’ movements were more efficient with shorter movement time and reduced error rate. As for the AR headset (HoloLens 2), Microsoft’s official documentation suggests that the focal distance is around 200 cm (2 m),^4^ which is longer than the fixation distance. Compared to the results from Swan et al. (2015) and Singh et al. (2018), which showed an increasing distance overestimation, the AR condition showed an initial distance overestimation that decreased as the target distance increased. Since the overestimation was attributed to the collimated display that renders the image much further than the observer’s fixation point, the trend towards underestimation in AR in the current study should be attributed to the rendered image being closer than the fixation, which was also consistent with the derived vergence offset of 0.18°. However, the discrepancy between HoloLens 2’s documented focal distance and the behavioral outcome implies that the oculomotor consequences of VAC could be contingent upon the magnitude of the difference between focal and fixation distance. A future study should systematically investigate this effect. The following discussion will focus on the effect of vergence offset and IPD deviation on distance perception given the current model setup.

### Interpupillary distance

**Fig. 6:**
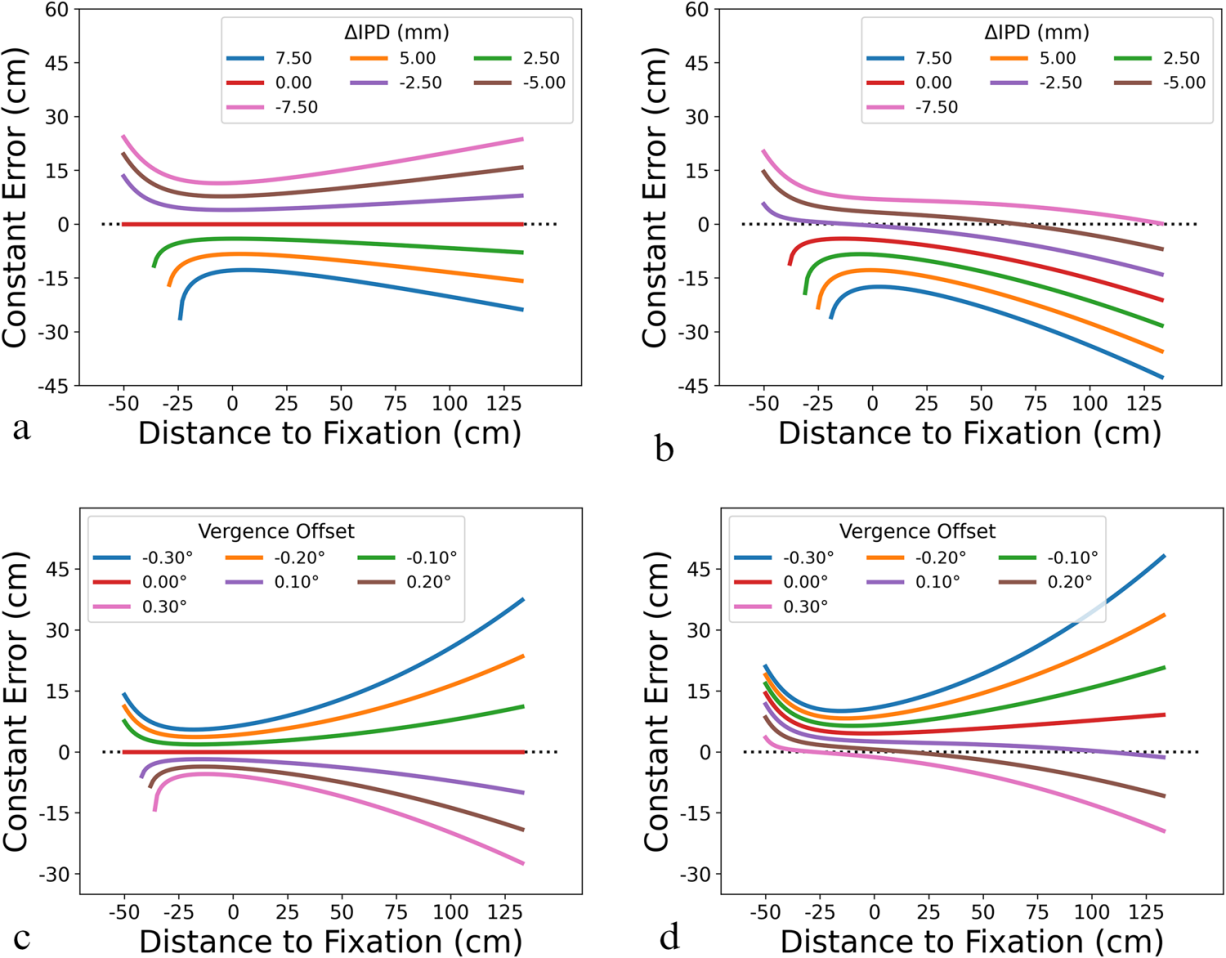
The model predicted constant errors mapped as a function of distance to the fixation point for different IPD deviations when: **a** there was no vergence offset and **b** when the vergence offset was empirically derived from the VR condition (0.22°), as well as for different vergence offsets when **c** there was no IPD deviation and **d** the observer’s IPD was the mean empirically derived IPD values from the VR condition (65.38 mm or ΔIPD =  − 3.13 mm). Following the experimental setup, the fixation point was placed 51 cm away from the observers (see 3.1.1 Model environment) and the simulation only used locations in front of the observers (i.e., distance to fixation > 51 cm). The black dotted lines mark a constant error of 0 cm

Figure 6 depicts some observations from the model and the fitted parameters. First, constant errors were computed without a vergence offset ($\beta_{{{\text{offset}}}}$ = 0°; Fig. 6a) and with the fitted vergence offset from VR ($\beta_{{{\text{offset}}}}$ = 0.22°; Fig. 6b) and plotted as a function of distance to the fixation point, where the fixation point was set to be 51 cm away from the observers. Different curves represent varying degrees of deviation of the HMD’s IPD from the observers’ ($\Delta {\text{IPD}} = {\text{IPD}}_{{{\text{HMD}}}} - {\text{IPD}}_{{{\text{par}}}}$), where changes in the observers’ IPD modulate the magnitude and sign of the constant error. For all scenarios, the constant error reaches singularity as the target gets closer to the observers (indicated by the missing values in each curve as the distance to fixation gets smaller) and the IPD difference affects the point at which the singularity occurs. When there is no vergence offset (Fig. 6a), a lack of IPD difference (red curve) yields a constant error of 0 cm, indicating that a lack of perturbation to the perceptual geometry enables veridical distance perception. As the observer’s IPD deviates from the HMD’s, changes in the constant errors appear to be symmetrical: positive ΔIPD results in negative constant errors and vice versa. This finding is congruent with studies that explicitly manipulated IPD and investigated its effect on targeted actions in the unmediated environment (Coats et al. 2014), where smaller IPD (positive ΔIPD) yielded underestimation and larger IPD (negative ΔIPD) yielded overestimation. Regardless of the direction of distance distortion, the increase in target distance corresponds to the increase in the magnitude of the distortion in the same direction.

When the fitted vergence offset from VR was used (Fig. 6b), the positive offset value shifted all the curves down and perturbed the symmetry observed in Fig. 6a. When the HMD’s IPD is equal to or greater than the observers’ (positive ΔIPD), the distance underestimation persists and the curves are still unimodal, yielding a local maximum representing the minimum amount of distortion to the perceived depth. In contrast, when the observers’ IPD is greater than the HMD’s (negative ΔIPD), the curves become strictly decreasing and the overestimation could transition to underestimation as the target moves away from the fixation point. More interestingly, the ΔIPD modulates the rate at which depth compression occurs. For instance, comparing the curves corresponding to −0.75 (pink) and 0.75 (blue) ΔIPD, the former’s slope after its corresponding inflection point is shallower than the latter’s slope passing its maximum. Although a similar trend can also be observed in Fig. 6a, the additional vergence offset amplifies this effect.

Overall, the above analysis showed that if VAC is unavoidable in the design of HMDs, to minimize the effect of vergence offset on distance perception in a virtual environment, one could intentionally incorporate a constant IPD deviation such that the magnitude of depth distortion could remain stable as the relative location of the target and fixation point changes. That is, the headset’s IPD value could be intentionally set to be smaller than the user’s actual IPD to minimize the effect of distance distortion due to VAC.

### Vergence offset

Then, regarding the effect of vergence offset, Fig. 6c illustrates the scenario in which the HMD’s and observer’s IPDs are identical and Fig. 6d shows when the observer’s IPD was the mean IPD from the VR condition (65.38 mm; i.e., ΔIPD = −3.13 mm). Similar to the effects of manipulating the IPD difference, changing the vergence offset also created symmetrical curves, where negative vergence offset consistently produced overestimation and positive offset produced underestimation. This similarity is expected given the perceptual geometry. Specifically, the vergence angle $\phi$ can be expressed as a function of IPD given the fixation distance D:

$$\phi = 2\arctan \frac{{{\text{IPD}}/2}}{{\text{D}}}$$

Or:

$${\text{IPD}} = 2{\text{D}}\tan \frac{\phi }{2}$$

To identify the equivalent of changes in vergence angle, $\beta_{{{\text{offset}}}}$ in changes in IPD, the following relationship can be established:

$$\Delta {\text{IPD}} = 2D\tan \frac{{\phi + \beta_{{{\text{offset}}}} }}{2} - 2D\tan \frac{\phi }{2}$$

Because

$$\tan \frac{{\phi + \beta_{{{\text{offset}}}} }}{2} = \frac{{\tan \frac{\phi }{2} + \tan \frac{{\beta_{{{\text{offset}}}} }}{2}}}{{1 - \tan \frac{\phi }{2}\cdot\tan \frac{{\beta_{{{\text{offset}}}} }}{2}}}{ }$$

And with the small angle ($\left| {\beta_{{{\text{offset}}}} } \right| \le 1^\circ$) approximation:

$$\tan \frac{{\beta_{{{\text{offset}}}} }}{2} \approx \frac{{\beta_{{{\text{offset}}}} }}{2}$$

The relationship can be simplified as

4 $$\Delta \,{\text{IPD}} \approx \frac{{4 \beta_{{{\text{offset}}}} D}}{{ - \beta_{{{\text{offset}}}} \sin \,\phi + 2\cos \phi + 2}}$$

**Fig. 7:**
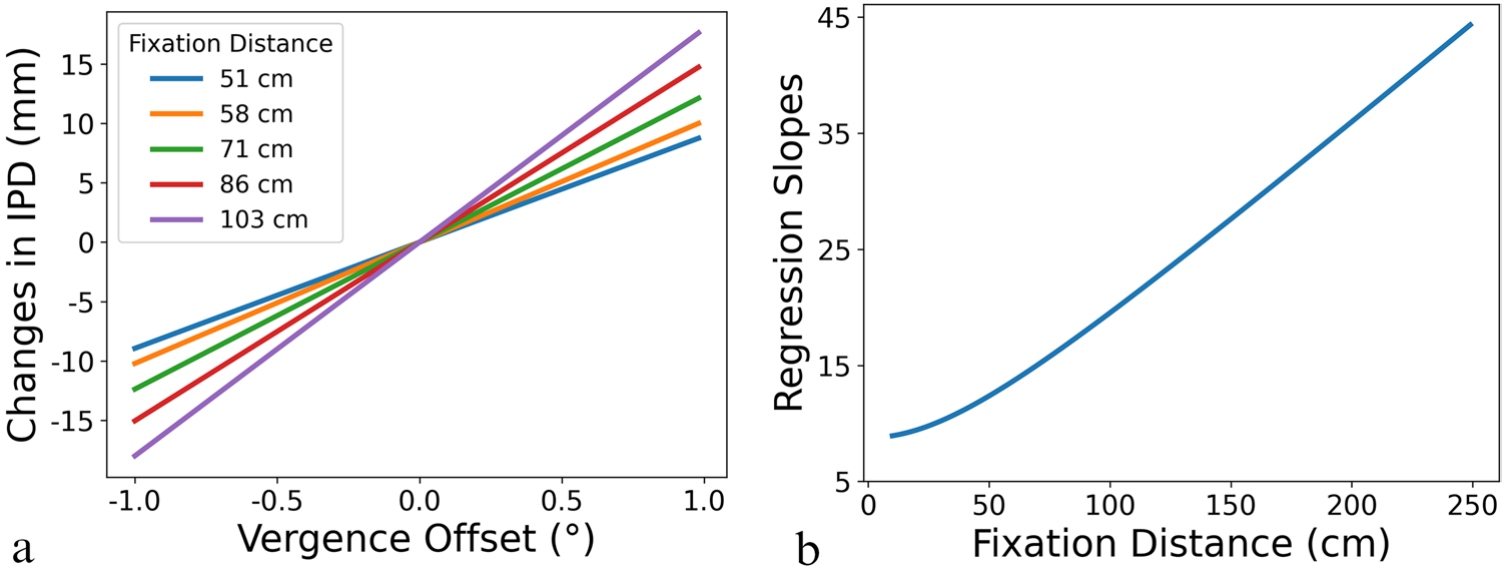
**a** A depiction of how changes in vergence offset, $\beta_{{{\text{IPD}}}}$, would translate to the equivalent change in IPD, $\Delta {\text{IPD}}$, for different fixation distances. **b** The regression slopes for the relationship between $\beta_{{{\text{IPD}}}}$ and $\Delta {\text{IPD}}$ for different fixation distances

Using the present study’s geometry (fixation distance D = 71.42 and the rendered fixation angle of $\phi$ = 5.01°),^5^ Fig. 7a (green line) demonstrates the relationship between $\beta_{{{\text{offset}}}}$ and $\Delta {\text{IPD}}$. Noticeably, although Eq. (4) indicates a nonlinear relationship between the two variables, it is apparent from Fig. 7a that a linear function may be sufficient. Using linear regression, the following relationship can be established ($r^{2}$ = 1.00):

$$\Delta \,{\text{IPD}} = 12.49 \beta_{{{\text{offset}}}} - 0.0016$$

However, as Fig. 7a also indicates, the location of the fixation point dictates the slopes between $\beta_{{{\text{offset}}}}$ and $\Delta {\text{IPD}}$. In fact, as shown in Fig. 7b, the changes in slopes do not follow a linear trend, especially when the fixation point is closer to the observers.

Overall, this analysis suggests that, even though perceptual geometry may entail the interconnection between IPD and the vergence offset, their relationship does not remain constant as the observers move their fixation point. In other words, although the ${\text{IPD}}_{{{\text{par}}}}$ and $\beta_{{{\text{HMD}}}}$ may contribute to the same variance in the behavioral results in model fitting, they should be treated separately on a conceptual and implementation level, as one cannot adequately capture the effect of the other. In fact, as the model comparison shows, versions with a single parameter (i.e., version 3 with only individual IPDs and version 4 with a single vergence offset) cannot account for the variance in the data as well as having both parameters—as in the baseline version. Nevertheless, acknowledging their interconnectedness may help to address the issue of low between-subject variability in the fitted ${\text{IPD}}_{{{\text{par}}}}$. That is, it was likely that the fitted vergence offset took away a portion of the variance that could have been attributed to the individual difference in ${\text{IPD}}_{{{\text{par}}}}$. To address this issue, future studies should explicitly measure the participants’ IPD and perturb its relationship with the ${\text{IPD}}_{{{\text{HMD}}}}$.

Finally, referring to Fig. 6d, when the IPD was the empirically derived mean (ΔIPD = −3.13 mm), the constant errors for different vergence offsets were mostly positive, which is congruent with the trends observed in Fig. 6a. Similar to the effect of ΔIPD, as the vergence offset increases from negative to positive, the patterns of the constant error changed from unimodal and convex to strictly decreasing, and the slopes of the curves after the inflection point varied as a function of the magnitude of the offset: The greater the offset, the steeper the slope.

Given these observations, an alternative solution to VAC could be proposed. Specifically, because the vergence offset is constant for a specific device, the proposed model could be used to predict the perceptual distortions resulting from the offset. Then, to eliminate the distortions, an inverse transformation could be applied to the rendered virtual 3D environment on an algorithmic level (e.g., increase the rendered distance in the 3D environment so that the perceived distance is not compressed). In fact, a previous study (Peer and Ponto 2019) quantified the amount of depth compression in VR using a blind throwing task and applied the inverse of this compression (“warps”) to the rendered 3D environment as a vertex shader. Results showed improvement in distance judgment after warping. The warps that Peer and Ponto (2019) devised were solely based on the linear relationship between target distance and constant errors, which, as shown in the present study, may not be adequate in capturing the curvilinear relationship between the two factors. In other words, the mechanistic description of the influence of VAC on distance perception in MR would provide a generalized approach to alleviate perceptual distance distortions in MR. Future user studies should evaluate the validity of the proposed model and explore whether applying the inverse geometrical transformation could help to ameliorate and/or eliminate depth distortions in MR.

### Limitations

The current study provides an alternative interpretation of distance distortions in MR solely based on the perceptual geometry that underlies stereoscopic vision. Admittedly, MR systems introduced many additional perturbations to the human visual system that could affect distance perception in peri-personal space. For VR, these perturbations in the present study (and most VR interactions) include a disembodied and non-articulating hand (Gonzalez-Franco et al. 2019; Mohler et al. 2010), the size and minor orientation differences between the participants’ physical hand and the virtual hand (Linkenauger et al. 2013), and the temporal delay between movement and rendering of the movement (Warburton et al. 2022). Similarly, for AR, although the participants used their physical hands to interact with virtual objects, issues such as the inappropriate occlusion of the hand (Bingham et al. 2001) and spatial drift (Miller et al. 2020) could also impact the measured motor performance in the current task. Furthermore, other device-specific factors, such as the headset’s field of view (Masnadi et al. 2021), weight (Buck et al. 2018), and screen resolution (Ryu et al. 2005) could also impact performance. In this context, although the current study only focused on one element of the myriad of perturbations that MR generates, the proposed model could still account for between 40 to 60% of the variance in the data. As previously mentioned, additional user studies are necessary to further validate the model and, more importantly, evaluate the potential of applying an inverse geometrical transformation to remediate depth distortions in MR.

Additionally, the movement endpoint was extracted based on a hard velocity cutoff that did not take into consideration the potential secondary, discrete adjustments and reversals that the participants could engage in to achieve higher movement accuracy. As mentioned previously, the targeted manual movement could be parsed into an initial (primarily) ballistic phase based on visual information and motor planning and a subsequent online correction phase where proprioceptive and visual feedback can be used to attain greater endpoint accuracy (Elliott et al. 2010). In the context of the current study, a hard velocity cutoff was used to ensure that the endpoint reflects the end of the primary movement which likely is more reflective of the perceived location of the target location based on the available visual information. In the context of human-computer interaction, however, users could rely on the visual feedback around the endpoint to perform additional corrective movements that entail reacceleration and movement reversal to arrive at the target location. While a companion study to the current work used a detailed kinematic analysis to demonstrate that the participants engaged in online corrections in VR (Wang et al. in preparation), the current kinematic analysis method did not account for the discrete adjustments. Future studies that explore the remediating effect of the inverse geometrical transformation could also factor in these discrete adjustments to examine whether the corrected depth rendering could help to reduce the need for such adjustments.

## Conclusion

Overall, the current study demonstrated that the VAC, a technical challenge that has been hampering the development and use of MR devices for decades, could potentially be geometrically modeled as a single offset in the vergence angle. Based on this model, suggestions were made to mitigate the effect of VAC, such as introducing intentional offset between the user’s and the headset’s IPD values to reduce distance perturbation, as well as adding an inverse spatial transformation to the rendering process to offset the effect of VAC on distance perception. Admittedly, there are elements to this geometrical description that require further investigations, such as introducing a more methodical investigation of the effect of IPD deviations, generalizing the finding to different target locations that are not limited to the sagittal plane on a single elevation, and investigating the relationship between the vergence offset and the headset’s focal distance. Continuing this line of inquiries will not only provide more insights into the fundamental functions of the human visual system but, more importantly, shed light on the potential development direction for MR-based applications.

## Data Availability

The datasets generated during and/or analyzed during the current study are not publicly available due to privacy concerns but are available from the corresponding author upon reasonable request.

## Footnotes

1 The term mixed reality (MR) is used following the virtuality continuum framework proposed by Milgram and Kishino (1994).

2 For consistency, the model description uses the following notation scheme: Upper case letters represent variables in the physical/rendering space (e.g., constants and 3D points). Lower case letters represent perceptual variables. Greek letters represent angular values. Letters with a ^ represent perceptual variables and those derived using the model.

3 Based on an email from Marty Banks to the Vision List. See https://visionscience.com/pipermail/visionlist_visionscience.com/2021/005947.html.

4 See https://learn.microsoft.com/en-us/windows/mixed-reality/design/comfort.

5 Given eye height of 50 cm, and that a fixation point is 51 cm away from observers, the fixation distance can be computed using the Pythagorean theorem ${\text{D}}=\sqrt{{50}^{2}+{51}^{2}}=71.42$ cm. Then, given the HMD’s IPD of 62.50 mm, the vergence angle is $\phi =2{\text{arctan}}\frac{6.25/2}{71.42}=5.01^\circ$. Other fixation distances presented in Fig. 7 were derived using different distances between the fixation point and the observers (i.e., 11, 31, 71, and 91 cm), instead of 51 cm.
